# Causal factors concerning the texture of French fries manufactured at industrial scale

**DOI:** 10.1016/j.crfs.2024.100706

**Published:** 2024-02-21

**Authors:** R.G.M. van der Sman, Esther Schenk

**Affiliations:** a Wageningen Food & Biobased Research, Wageningen University & Research, the Netherlands; b Lamb-Weston/Meijer V.O.F, the Netherlands

## Abstract

In this paper, we review the physical/chemical phenomena, contributing to the final texture of French fries, as occurs in the whole industrial production chain of frozen par-fried fries. Our discussion is organized following a multiscale hierarchy of these causal factors, where we distinguish the molecular, cellular, microstructural, and product levels. Using the same multiscale framework, we also discuss currently available theoretical knowledge, and experimental methods probing the relevant physical/chemical phenomena. We have identified knowledge gaps, and experimental methods are evaluated in terms of the effort and value of their results. With our overviews, we hope to give promising research directions such to arrive at a multiscale model, encompassing all causal factors relevant to the final texture. This multiscale model is the ultimate tool to evaluate process innovations for effects on final textural quality, which can be balanced against the impacts on sustainability and economics.

## Highlights

• Causal factors determining texture of finished French fries are reviewed.
• Causal factors are discussed using a multiscale framework.
• Knowledge sources for causal factors, and their complexity are listed.
• Measurement/Imaging techniques for identification of causal factors are reviewed.

## Graphical abstract

**Figure:**
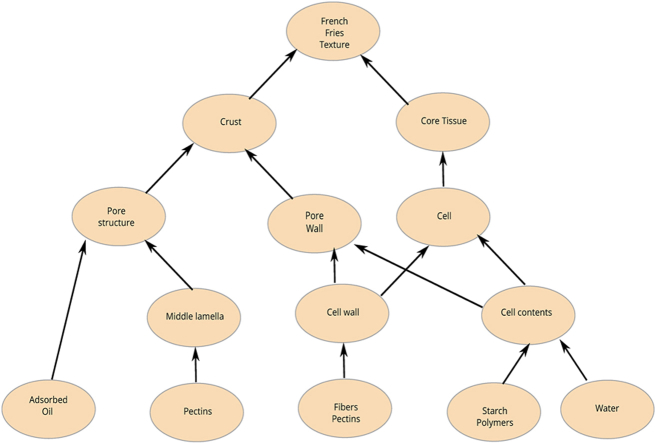

## 1 Introduction

In our recent review paper, we have argued that for optimization of the quality of frozen vegetables, one has to assess the impact of the complete production chain (Van der Sman, 2020). Frozen French fries are a special case in the class of frozen vegetables, as they attain their final quality via frying in oil, instead of boiling in water. Moreover, the desired textural attributes are significantly different from other vegetables. French fries are desired to have a crispy crust combined with a soft, mealy core (Miranda and Aguilera, 2006). Nevertheless, there are sufficient similarities with other frozen vegetables, as French fries are also made of plant tissue, and they are also subject to blanching before freezing to inactivate enzymes. However, in contrast to most vegetables, potatoes contain a large portion of starch, enabling French fries to attain the typical texture of their crust and core. Because of the differences in texture and processing steps, frozen French fries justify a specialized review on the optimization of quality, i.e. texture. How to measure textural properties of French fries (Du Pont et al., 1992) (Jarén et al., 2016) (Li et al., 2020a), and the impact of processing on texture (Dourado et al., 2019) (Arefi et al., 2022) have been reviewed already several times. However, we think much can be gained via considering an intermediate link, which describes how texture relates to physical/chemical properties, and how these factors are influenced via processing, as advocated by (Van Koerten et al., 2015). Furthermore, there are only a rare number of papers considering the impact of various unit operations from the complete industrial production chain on final quality of French Fries (Touffet et al., 2020) (Li et al., 2020b) (van der Sman and van den Oudenhoven, 2023).

Consequently, in this paper, we review the physicochemical causes of the textural changes, and how they are mediated via the unit operations from the production chain. The paper is not intended to provide a critical review of all papers, but to provide a unique synergetic view of how different physical-(bio)chemical processes contribute to the texture of French fries. We will assemble hypotheses from different papers, often originally applied to other vegetables or non-edible soft matter.

First, we give a detailed account of the important textural attributes, as commonly assessed by a trained sensory panel. This is combined with a discussion on physical measurements, that are intended to probe physical texture properties directly, assuming they are representative of the perceived sensory texture. Subsequently, we discuss the potential (bio)physical causes attributed to the texture of French fries. We order these attributes according to a multiscale hierarchy of structures, as inspired by the approach of (Waldron et al., 1997), which is applied to cooked vegetables and potatoes. This multiscale approach is attracting attention, such as in the relevant related fields of fruit maturation (Segonne et al., 2014) (Cáez-Ramirez et al., 2015) and fruit mechanics (Li and Thomas, 2016), and fruit/vegetable drying (Welsh et al., 2018) - where similar physicochemical phenomena are happening as in French fries processing like pectin solubilization, pectin methyl esterase (PME) activity, and cell separation. In the multiscale structure for French fries proposed in this review, we make an explicit distinction between crust and core, as they give different contributions to texture. Furthermore, in addition to having a multiscale structure, these physical/biochemical factors form an intricate web of causal relations. We will present this visually through a causal network diagram to provide the reader with a clear and comprehensive overview.

The precise relationships between texture and (bio)physical causes, as well as their influence by processing, remain not fully understood. Much of the modelling of texture is currently very empirical (Chen and Opara, 2013). Hence, we view it would be helpful to provide an overview of 1) quantitative models describing these relevant (bio)physical causes, and 2) experimental methods probing these physical causes. To aid the reader in choosing appropriate methods from the myriad of measurement techniques, we have scored them in terms of 1) effort to perform the measurement (investment, time), and 2) estimated value of the obtained results (from our perspective).

Via measurements on the relation between processing and these physical causes, we think one can get better knowledge on how to steer the texture of French fries in the desired direction. We exemplify that via a discussion of alternative processing methods, on how they target these physical causes and thus can help steer texture.

**Fig. 1:**
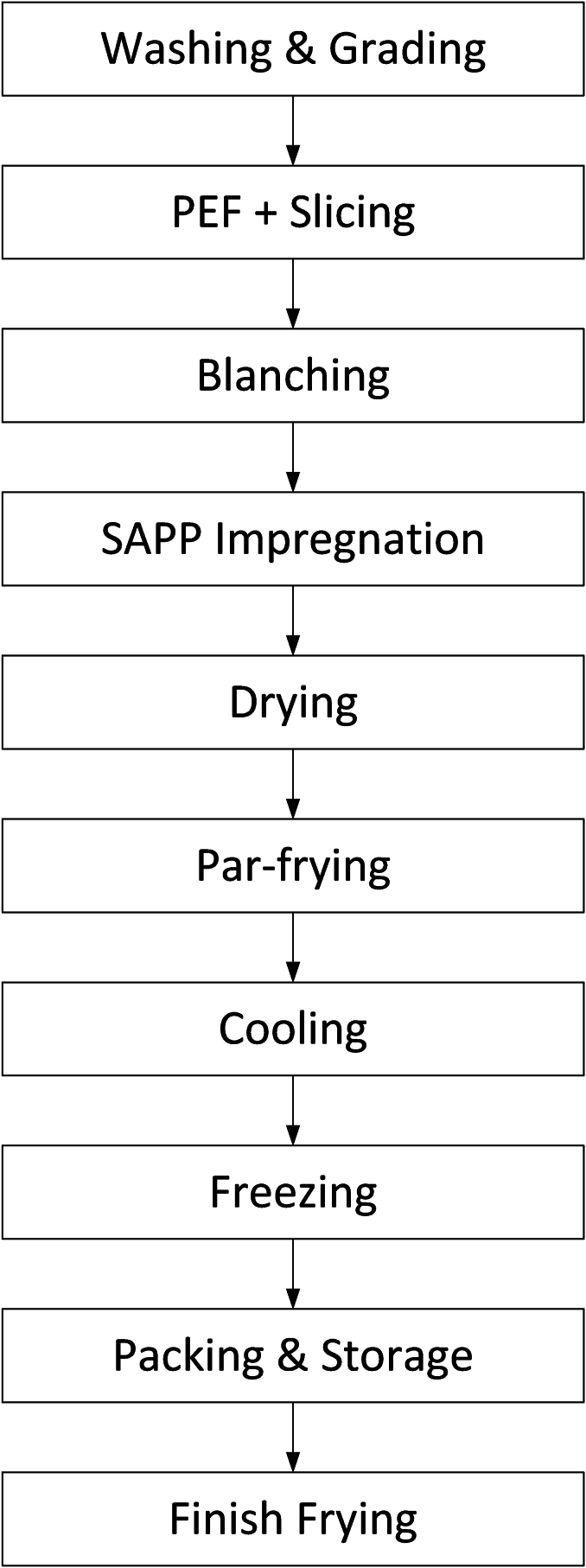
Simplified representation of the industrial production chain of frozen par-fried French fries. They are stored in the frozen state, and at the point of consumption, they will be finish-fried.

Regarding processing, we focus on French fries, that are handled at an industrial scale, as discussed previously (van der Sman, 2018) (Van der Sman, 2020). In Fig. 1 we show a sketch of the unit processes involved in the industrial processing of French fries, with the final step of finish-frying typically done at the consumer or the point of sale. We have to remind the reader that, many studies in literature investigate the direct frying of raw potatoes, which is not in line with industrial practice (Miranda and Aguilera, 2006) (Moyano et al., 2007), where potato strips are first blanched, par-fried, and frozen, before finish-frying. Hence, care must be taken for the translation of their findings to industrially manufactured French fries.

## 2 Texture of French fries

The texture of a French fry has a characteristic texture contrast: the crust is crispy, while the core has the texture of a cooked potato, with a distinct mealiness. Furthermore, the internal texture of the core is also characterized by a certain firmness. Cooked potato texture can also be described in terms of dryness (Jarvis et al., 1992), which is related to the degree of cell separation. Mealy cultivars are attributed to softness/dryness/floury/mealy, while waxy cultivars are attributed to be moist and firm. Distinguishing between crispiness and crunchiness is essential (Luyten et al., 2004). Crispiness is associated with the sound and force of the initial bite using incisors, while crunchiness is related to the sound and force during multiple chewing motions between molars. Crispy materials can show complete failure during a single biting event, while crunchy materials require multiple bitings for complete failure (Luyten et al., 2004).

Undesirable features may appear in the internal or external texture of French fries. The core, for instance, might exhibit ’hollowness’ with large cavities (Yin and Panigrahi, 2004), or beneath the crust, blisters can occur (Costa et al., 2001), often resulting from the collapse of the crust during pre-drying (Botero-Uribe et al., 2017). Blisters and cavities often have sizes larger than potato cells, and we explicitly distinguish them from the ”desired” smaller pores in the crust. Cavities and blisters are undesired, as they can draw in frying oil, and subsequently influence the sensation of crispiness (Vauvre et al., 2014).

Below, we will review the physicochemical causes of these texture developments during processing. A sensory panel typically scores a range of texture attributes when assessing French fries. These attributes, along with the measurement method (sensory panel and/or physical measurement) and the possible physicochemical cause, are listed in Table 1, Table 2, Table 3 and will be discussed in the following section.

**Table 1:** Characteristics of external texture.

Characteristic	Method(s) to quantify characteristic	Possible cause(s)
Crispiness	Trained sensory panel	Porosity (size and wall thickness)
	Acoustics + Texture analyzer	Brittleness + Crust corner compactness
		Crust structure + Crust thickness
		Core hardness (sound damping)
Crunchiness	Trained sensory panel	Porosity (size and wall thickness) + Brittleness
	Acoustics + Texture analyzer	Crust corner compactness + Crust structure
Blisters	Image analysis	Case hardening
Crust thickness	Image analysis	Moisture loss and moisture gradient

**Table 2:** Characteristics of internal texture.

Characteristic	Method(s) to quantify characteristic	Possible cause(s)
Firmness internal	Trained sensory panel	Cell wall strength + Cell separation
	Texture analyzer	Gelatinization/swelling starch
		Moisture content/gradient
Graininess	Trained sensory panel	Starch retrogradation
Mealiness	Trained sensory panel	Cell wall strength
	Rheometer	Cell separation
Hollowness (large cavities)	Image analysis	Crust thickness + Crust strength/hardness
		Cell separation + Cell wall strength

**Table 3:** Characteristics of total texture.

Characteristic	Method(s) to quantify characteristic	Possible cause(s)
Firmness total	Trained sensory panel	Cell wall polymer chemistry
	Texture analyzer	pectins/hemicelluloses/lignin
Contrast inside outside	Sensory panel/Texture analyzer	Crispiness versus Firmness internal
Chewiness	Sensory panel/Texture analyzer	Crispiness and Firmness internal
Dryness	Sensory panel	Cell separation, Saliva Uptake, Intactness cells
	Water holding capacity	Cell wall strength, Starch gelatinization

## 3 Physicochemical causes to textural changes

As follows from our discussion there is a myriad of physical/(bio)chemical factors affecting the internal and external texture, many of which influence each other. Due to the shear number of papers discussing all these factors, it is not possible to give all of them a critical review. Instead, we like to provide the reader with a good overview of the causal relationships between these factors. In addition to our discussion below, we have constructed two visual representations of the causal networks of these physical/(bio)chemical factors, as shown in Fig. 3, Fig. 4. There, the causes are depicted as blue ellipsoids, which are connected via either a) blue arrows indicating positive correlations, or b) red barred arrows indicating negative correlations. Some of the basic causes can be controlled via processing conditions. These cases are indicated via their connection to unit operations present in the industrial production chain. As an alternative to a critical review of papers, we provide a table of indicating key scientific papers discussing hypotheses concerning these physical/(bio)chemical factors, often from a microstructural perspective. Hence, in Table 4 we have indicated the discussed topic, and the experimental microstructural analysis techniques. The abbrevations are explained in the section on measurements.

**Fig. 2:**
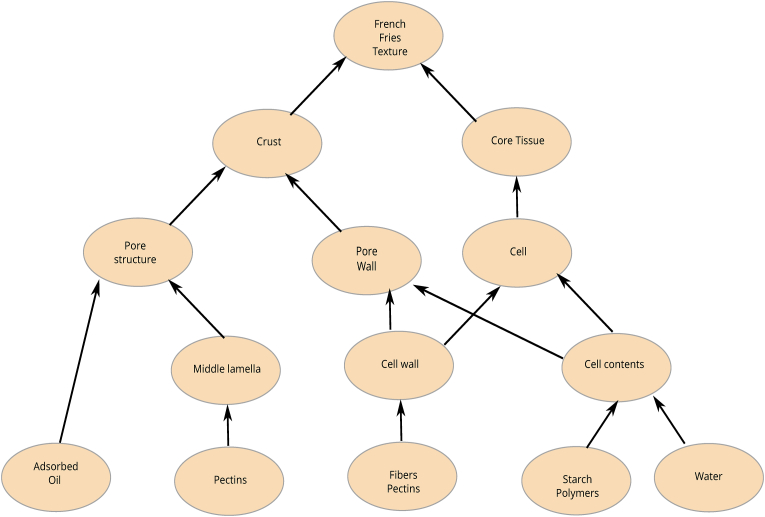
Multiscale representation of structures contributing to texture of French fries, inspired by the approach of (Waldron et al., 1997). The crust structure is approximated by a porous material, with a glassy matrix of starch and cell wall material. The core is viewed as a cellular structure, with gelatinized starch within the cell, and it has some degree of cell separation due to pectin degradation.

**Fig. 3:**
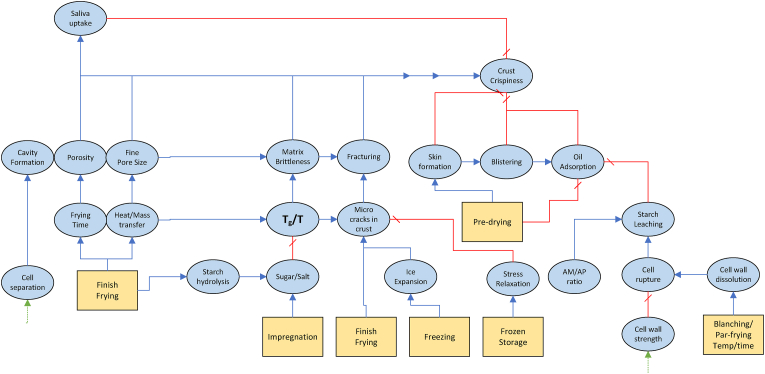
Causal network for external (crust) texture. Blue arrows indicate positive correlations, while red barred arrows indicate negative correlations. (For interpretation of the references to color in this figure legend, the reader is referred to the Web version of this article.)

**Fig. 4:**
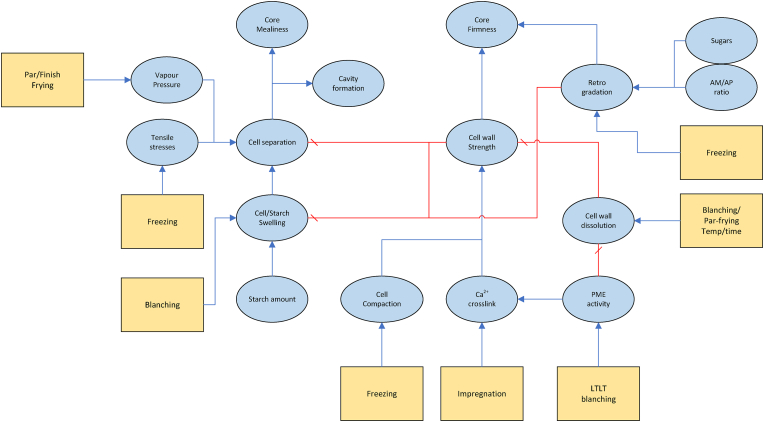
Causal network for internal (core) texture. Blue arrows indicate positive correlations, while red barred arrows indicate negative correlations. (For interpretation of the references to color in this figure legend, the reader is referred to the Web version of this article.)

**Table 4:** Critical papers providing hypotheses based on microstructural analysis.

Ref	Technique	Causal Factors addressed
Van Loon et al. (2007)	CSLM	Skin formation/Collapse
Van Koerten et al. (2015)	XRT	Crust structure
		Crispiness
Vauvre et al. (2014)	XRT + CSLM	Oil adsorption
		Cavity formation
		Crust structure
Gouyo et al. (2021a)	XRT	Crust structure
		Crispiness
Kalogianni and Papastergiadis (2014)	SEM	Crust structure
		Crust shrinkage/Collapse
Jarvis et al. (1992)	Swelling volume via Argon bubble	Cell Separation
		Cell/Starch swelling
Ormerod et al. (2002)	Microscopy	Starch swelling
		Cell separation
Ding et al. (2022)	Microscopy/FTIR/XRD/DSC	Cell/Starch swelling
		Cell wall crosslinking
Ross et al. (2011)	Antibody Microscopy/FTIR	PME actvity
		Core structure
		Cellular adhesion

The above mentioned physical/(bio)chemical factors relate to phenomena happening in structures of the French fries having distinct length scales. This multiscale structure representation of both crust and core are depicted in Fig. 2, which was inspired by the original work of (Waldron et al., 1997). We will use this multiscale representation to structure our discussion of the physical/(bio)chemical causes of textural changes in crust and core, as follows in the next two sections.

### 3.1 External texture

#### 3.1.1 Crust development: crust thickness & moisture transport

The crispy crust develops during finish-frying at the point of consumption. Moisture is rapidly lost from the crust region through frying, facilitated by the high heat transfer in the agitated oil, resulting from the violent release of steam bubbles from the potato strip. As diffusive moisture transport in the core is limited, the moisture is primarily lost from the crust region. At intensive heating as during frying a sharp evaporation front (at 100^*o*^C) moves inward (Sam Saguy and Dana, 2003) (Miranda and Aguilera, 2006), and, consequently, a clear transition between the crust and core structures will develop (Van Koerten et al., 2015).

Studies have shown that crust thickness is linear with the square root of frying time, indicating diffusion-driven behavior (Farkas et al., 1996) (Gouyo et al., 2020). Crispiness is related to the moisture content of the outer crust. However, it is concluded that for overall texture the rate of moisture loss (governed by heat transfer coefficient) is more important than total moisture loss (Gouyo et al., 2020). The rate of moisture loss will determine the moisture gradient in the crust region.

We anticipate an impact of par-frying on crust crispiness, as the increase in crispiness with finish-frying time is diminished when par-frying time is reduced (Sanz et al., 2007). Simply adding the two frying times does not yield similar results. A likely cause is the action of freezing on the microstructure. The crust region remains unfrozen for a while during freezing, because of its lower moisture content and higher salt/sugar concentration (van der Sman, 2023a). While the core freezes and expands, it imparts mechanical damage to the non-expanding unfrozen crust. This phenomenon is caused by the freezing point depression induced by 1) the moisture removal during par-frying and 2) added sugars/salts during the impregnation step after blanching. The freezing core expands because ice occupies more volume than liquid water, leading to tensile stresses, especially at the interface between crust and core (Tremeac et al., 2007) (van der Sman and van den Oudenhoven, 2023). During the subsequent frozen storage, the crust region will eventually freeze as well. However, the tensile stresses will be locked in and relax away only very slowly (van der Sman and van den Oudenhoven, 2023).

#### 3.1.2 Crust structure: porosity and collapse

Due to the strong moisture loss, the cells in the crust will shrink and dry out, until the crust material enters the glassy state. Via the moisture loss, the hardness/brittleness of the crust increases (Van Koerten et al., 2015, 2017). Consequently, tensile stresses develop during frying, leading to porosity development in the crust region, but also to the growth of cavities, just beneath the crust (Lewicki and Pawlak, 2005) (Gouyo et al., 2021a).

Measurements with texture analysers and of acoustics show a good correlation between crispiness and the number of peaks in force/displacement or acoustic emission (Dogan and Kokini, 2007) (Saeleaw et al., 2012) (Van der Sman et al., 2018). Crispiness is shown to correlate to porosity, the number of small pores, and the ratio of pore wall thickness to pore size (*t*/*R*) (Dogan and Kokini, 2007). Mechanical energy for deformation is also a function of this ratio, and the strength of the matrix (elastic modulus).

The amount of porosity/cavities is dependent on viscoelastic properties, which are strongly moisture dependent (Vauvre et al., 2014). Also, it is shown that pre-frozen fries show little volume shrinkage/collapse, and thus develop higher porosity (Gouyo et al., 2021a), while frying potato strips from the raw state gives significant volume shrinkage/partial collapse (Vauvre et al., 2014) (Gouyo et al., 2021a). It is expected that the cell wall structure remains intact, but it will be wrinkled/convoluted due to shrinkage (via moisture loss) (Costa et al., 2001). The crust is expected to show largely a closed pore structure (Vauvre et al., 2014).

The features of the porous structure appear to depend on the heat/mass transfer rate, frying time, and temperature (Van Koerten et al., 2015) (Gouyo et al., 2021a). It is said that the heat/mass transfer rate determines the pore size, with fast transfer rates generating many small pores, and slow transfer rates generating fewer, but larger pores (Van Koerten et al., 2015). The higher the rate of moisture loss, the higher the texture contrast between crust and core. At similar moisture removal, a higher frying temperature (related to the rate of moisture loss) leads to higher porosity. Porosity can only develop if a critical temperature is reached (where the shrinkage cannot keep up with moisture removal). This critical temperature is related to the glass transition temperature *T*_*g*_. At slower heat/mass transfer, the crust is a considerable amount of time in the rubbery state, which allows the crust to compact itself, also leading to a less extended crust region (Van Loon et al., 2007). The sooner the crust reaches the glassy state, the more porosity is developed (for a given frying time).

Crispiness is also investigated in fried, potato starch snacks, showing the importance of soft fillers to crispiness (van der Sman et al., 2021) (Van der Sman et al., 2018). In the case of snacks, potato flakes are often used as soft fillers. As potato flakes are loose, cooked potato cells, this finding can imply that some degree of cell separation in the crust can help create crispiness.

If cell wall material in the crust region is sufficiently weak, the cells can burst during par-frying - leading to the leaching of starch into the crust microstructure, where it can form a film/coating, thus preventing/reducing oil adsorption. If swelling of cells during blanching is too extensive (due to weak cell wall), the cells can also rupture (Reeve, 1967).

Cavities larger than potato cells are observed just beneath the crust (Kalogianni and Papastergiadis, 2014; Vauvre et al., 2014). It is assumed to occur due to steam pressure build-up, leading towards cell separation (Kalogianni and Papastergiadis, 2014). Weak spots, where cavity formation is likely to happen, are likely already generated during freezing, where tensions/stresses are concentrated at interface crust/core leading to cavity formation (Vauvre et al., 2014).

The drying step in between blanching and par-frying can cause skin formation at the surface (also known as case hardening (Gulati and Datta, 2015)), which can act as a barrier for moisture loss/oil adsorption (Botero-Uribe et al., 2017). However, this skin will decrease crispiness and promote blistering (Van Loon et al., 2007). The skin prevents steam from escaping during finish-frying, and thus vapour pressure builds up underneath the skin, creating a large pocket (Van Loon et al., 2007) (Kalogianni and Papastergiadis, 2014) (Vauvre et al., 2014), which might collapse after removal of the potato strips from the fryer, if the surrounding material is not in the glassy state (Costa et al., 2001). It must be noted that in the study (Kalogianni and Papastergiadis, 2014) non-frozen potato strips are fried. In contrast, another study (Gouyo et al., 2021a) shows that fried pre-frozen strips showed much less collapse of the skin, leading to a highly porous skin, with small pores. With microscopy, it is shown that crust structures with more flattened cells have more crispiness, compared to structures with still round cells (Van Loon et al., 2007). If cell walls are sufficiently weak and are dehydrated fast, they will flatten during finish-frying. Cell flattening is not so much a result of drying/par-frying.

#### 3.1.3 Brittle/hardness

The crispiness of the crust is perceived during eating. If the crust is near or in the glassy state, energy dissipation (as a reaction to the work input of mastication) will occur mainly via fracturing (Luyten et al., 2004; Vliet et al., 2007), leading to perception of a brittle texture. Crispiness is promoted by the hardness of crust material (as modulated by moisture content), porosity, small pore sizes, and thin pore walls. However, it is said that fracturing cannot account for all energy dissipation, also the viscoelastic relaxation provides dissipation (Ross and Scanlon, 2004). Also, heterogeneity contributes to crispiness (more randomness in sound emission) (Vincent, 1998). Crack-stopping elements, like pores and oil pockets, will modulate the crispiness/brittlenss, preventing complete failure during a single biting event (Vincent, 1998).

Later, we have shown that *T*_*g*_/*T* is also a good measure for mechanical properties of food materials (Siemons et al., 2020) (Siemons et al., 2022) (Van der Sman et al., 2022), indicating the central role of glass transition *T*_*g*_ in mechanical and textural properties. Following Gibson-Ashby, the porosity also influences the elastic modulus (Robin et al., 2010). Thus the potential collapse/shrinkage of the crust happening while it is still in the rubbery state will also influence the hardness/crispiness. It is expected that collapse can be less if the cell wall has higher strength. Hence, measures to enhance cell wall strength, as discussed in the next section of core texture, can also contribute to crispiness (Moreira et al., 2021) (Arefi et al., 2022).

Frequently, a strong correlation between crispiness and water activity has been observed (Arimi et al., 2010). It's crucial to note that water activity is an indirect measure of moisture content, with the latter being the actual governing parameter determining the glass transition temperature, *T*_*g*_. This relationship is evident in the Gordon-Taylor or Couchman-Karasz relations for *T*_*g*_.

The glass transition temperature of the crust can be modulated via leaching/impregnation of solutes before/during blanching, which is already common practice: leaching/impregnation of sugars is used to control the browning of fries during finish-frying. Sugars act as coplasticizers, next to water, affecting the *T*_*g*_. Also, salts, which can be added during impregnation to control taste, are thought to modify *T*_*g*_ (Yi See et al., 2023). However, we should note there is no consensus on the effect of salts on *T*_*g*_ (Farahnaky et al., 2009) (Van der Sman and Broeze, 2014a) (Yi See et al., 2023). It is shown that the *T*_*g*_ of the crust can be modified via a coating (Rahimi et al., 2017).

A sudden increase in the hardness of the crust is observed when the frying temperature exceeds 180^*o*^C (Van Koerten et al., 2015). It was attributed by the decomposition of sucrose into reducing sugars, which participate in Maillard reactions, and subsequently lead to crosslinking with potato proteins, and hardening of the crust.

#### 3.1.4 Oil and saliva uptake

Oil adsorption appears to affect crispiness. It does not change the mechanical properties of the crust but rather reduces the sound emission, via deflection of sound at the oil–air interfaces (Vliet et al., 2007). Hence, reducing oil adsorption can be a tool to increase crispiness. From a health perspective, there has been a lot of study into the oil adsorption by French fries.

Oil adsorption happens after the removal of the fries from the fryer (Vauvre et al., 2014). After finish-frying the crust is a porous layer with interconnected small pores, underneath large cavities. Oil will penetrate this structure, depending on its viscosity. As such, palm oil can solidify upon cooling (after being taken out of the fryer), which increases its viscosity, leading to less penetration (Gertz, 2014). The absorbed oil will affect the crispiness, via damping of sound, and softening of the crust material (similar to oil in dough).

A recent study shows a surprising effect of freezing on the oil adsorption of industrial-manufactured French fries (Touffet et al., 2020), whose observations also indicate a direct impact of freezing on the texture of crust and core. Due to ice expansion during freezing and frozen storage, mechanical stresses can arise. Tensile stresses will be particularly concentrated at the interface between crust and core. These stresses amplify the cell separation, that already happened during blanching. Moreover, for pre-frozen potato strips this expanded network of fissures provides a second mechanism pressure driven of oil transport: between the evaporation front (at boiling point), and ice melting front (at freezing point), there is a vapour pressure gradient, which can draw in oil into the inner core structure (Touffet et al., 2020).

It is also mentioned that the crispiness sensation is impacted by saliva uptake by the porous structure. Small interconnected cells lead to quick saliva uptake (Luyten et al., 2004).

### 3.2 Internal texture

#### 3.2.1 Core development

The internal texture is determined by the combined effects of the blanching step, par-frying, and finish-frying. The desired texture should resemble that of a cooked potato. Hence, we have mostly addressed the literature on cooked potato texture, which is also expressed in terms of mealiness/waxiness, and firmness.

Mealiness sensation can be related to cell separation, and viscoelastic properties, but also starch gelatinization/swelling leading to cell rounding (distention), contributing to stresses leading to separation (Singh et al., 2009). The other sensory attribute of firmness is more related to starch gelatinization/swelling/retrogradation, and cell wall strength (Liu and Scanlon, 2007). During freezing further damage to the cellular structure of the core can occur, due to the tensile stresses arising from the ice expansion (Van der Sman, 2020). Consequently, the cell separation can be amplified.

The softness of cooked potatoes is attributed to both cell separation and cell wall strength, while dryness is only related to cell separation (Jarvis et al., 1992). Waxy potato cultivars can show cell fracture and subsequent juice release when sliced, as opposed to mealy cultivars where cells remain more intact. Dryness sensation is attributed to the propensity of the potatoes to absorb liquid (saliva), as gelatinized starch can still take up some water, or the lack of juice release when cut/fractured (by teeth).

#### 3.2.2 Core structure

Cell separation is caused by pectin dissolution, vapour pressure build-up, and cell swelling due to starch gelatinization (Jarvis et al., 1992). Pectin is important as it glues the cells together in the middle lamellae. Cells are glued together via calcium crosslinks between the pectins in the middle lamella if they are low in methyl-esters. Pectin dissolution degrades thus the middle lamella, leading to cell separation. Starch gelatinization and pectin dissolution happen already during blanching. Another main objective of blanching is the inactivation of polyphenol-oxidase (PPO) enzyme (causing discoloration), which happens at T = 80–100^*o*^C, while pectin dissolution via *β*-elimination already starts at *T* > 60^*o*^C and if pH > 4.5.

Similar to carrots (Ando et al., 2019a), we expect that freezing treatments create a more open cellular structure, which improves moisture transport during further-processing like the finish-frying. Given the similarity between finish-frying and drying, one might expect that the freezing damage to potato tissue is beneficial for the crust texture in rendering a more porous structure. However, a more open cellular structure also enhances the transport of oil to the core (Touffet et al., 2020), which can counter the enhancement of crispiness.

#### 3.2.3 Cellular scale: cell separation

Stresses from starch swelling during gelatinization cause cell rounding, contributing to cell separation (Jarvis et al., 1992) (Jarvis, 1998). The extent of cell separation also depends on the solubilization of pectin during thermal processing (Vauvre et al., 2014).

Starch swelling represents a balance between moisture uptake during blanching and cell wall strength (Jarvis et al., 1992). However, another study suggests that cell wall weakening through pectin degradation is the more dominant factor influencing mealiness (Ormerod et al., 2002). This is supported by the observation that in cooked potatoes the texture in the low-starch pith is similar to surrounding tissue with higher starch content (Ross et al., 2011). The pectin dissolution impacts the cell wall strength (Botero-Uribe et al., 2017). Starch may not be fully gelatinized because of limited water availability, or the strength of the cell walls, which limits the swelling. The increasing vapour pressure during frying can expand cavities in between cells (the intercellular space at junctions) (Vauvre et al., 2014), which is also modulated by the dissolution of pectin in the middle lamella.

Turgor-generated stress appears to be concentrated at the edges of the cell faces if cells swell due to osmosis. It was expected that cell separation starts at the cell edges, and hence nature has strengthened these regions with extra crosslinks at the edges (Parker et al., 2001). Given the similarity between the swelling pressure of gelatinized starch and turgor pressure (Jarvis et al., 1992), similar mechanisms play a role in cell separation during the processing of French fries.

Core texture can depend on the botanical properties of potato cultivars. Of course, the mealiness depends on whether the cultivar is a mealy or waxy potato cultivar. Already decades ago, it was found that the dry matter content of potatoes is significantly correlated with internal texture (Jaswal, 1970) (Matsuura-Endo et al., 2002). High-specific-gravity potatoes show both a firm and mealy texture. The cell size is also related to texture: larger cells have less contact surface area per cell volume, and therefore they are easier to separate. Immature potatoes have poor mealiness (but a waxy texture). It is stated that often botanical properties of potatoes are correlated: mealy potatoes have more starch (dry matter), more amylose, larger cells, and weaker cell walls (easily solubilized) (Reeve, 1967). The higher amount of starch (dry matter) can also lead to higher swelling pressure, and in combination with weaker cells, this leads to enhanced cell separation (Matsuura-Endo et al., 2002). Also, French fry texture is correlated with the total amount of non-starch polysaccharides, which varies amongst cultivars (Tajner-Czopek et al., 2003). These non-starch polysaccharides entail celluloses/hemicelluloses, which contribute to the strength of cell walls.

#### 3.2.4 Molecular scale: cell wall/pectin chemistry

Low methyl-esterified pectin is particularly concentrated at the edges of cell faces, which adhere to other cells (Ross et al., 2011). This concentration of low methyl-esterified pectin allows for localized Ca^2+^ crosslinks, strengthening the edges of cell faces - and making the cell separation more difficult. It might be further strengthened by local ferulic crosslinks (Waldron et al., 1997; Parker et al., 2001).

Organic acids (like citric acid) or EDTA can act as chelators of Ca^2+^, preventing the formation of crosslinks or even causing their dissolution (Andersson et al., 1994) (Matsuura-Endo et al., 2002). (Waldron et al., 1997) also reported on the effects of ferulic crosslinks in vegetables on cell adhesion. These ferulic crosslinks are particularly strong near cell junctions, where cell separation will start. Some vegetables like beetroot are quite rich in ferulic acids. Potato can also have these groups, but it is expected to be more distributed over the cell wall (Parker et al., 2001). Crosslinking appears to limit the cell wall dissolution (Ding et al., 2022).

Beta-degradation of pectin depends on the degree of methylation (number of methyl-esters). At LTLT-blanching (low temperature, long time) one can activate PME (pectin methyl-esterase) enzyme, which removes these methyl groups, leading to less pectin degradation/cell separation (Verlinden and De Baerdemaeker, 1997), and thus the cell wall retains some of its strength (Ross et al., 2011). The action of PME also allows for the crosslinking of pectin with Ca^2+^, which increases cell wall strength - leading to higher firmness, and less starch swelling (Tajner-Czopek, 2003). One should mind that calcium ions promote beta-elimination of pectin if the PME enzyme is inactivated at the early stage of blanching (if the LTLT step is not applied) (Kunzek et al., 1999).

The texture of low specific gravity potatoes can be improved by Ca^2+^-assisted LTLT blanching, but they cannot increase the firmness of high gravity potatoes (Jaswal, 1970). Raw potatoes already have amounts of Ca^2+^ present in the tissue, that help with cell wall strengthening (Murayama et al., 2017). This amount in raw potatoes can be increased via increased use of fertilizers during growth. In some varieties, the endogenous calcium can be too low, and it has to be supplemented via impregnation before blanching (Ng and Waldron, 1997).

#### 3.2.5 Molecular scale: state of starch

The starch gelatinization can be limited by the restraining cell wall. Intentional crosslinking of cell wall restricts starch swelling and gelatinization even further (Ding et al., 2022). The amount of starch swelling depends on the number of phosphorous groups in potato starch (Karim et al., 2007). However, the phosphorus content is shown to be negatively correlated with the amylose content, which can restrict swelling if retrograded. Hence, these effects cannot be separated.

Depending on the amount of starch swelling, amylose can be leached out of the granule - which can be retrograded during cooling steps in the fry processing (Lewicki and Pawlak, 2005). However, one should mind that amylose leaching can be inhibited if swelling/gelatinization is incomplete due to a strong cell wall. Amylose retrogradation during cooling can lead to a decrease in cell volume. Amylose retrogradation happens at short time scales, during cooling and/or freezing - with a maximum retrogradation rate at 4^*o*^C (Reeve, 1967).

Retrogradation of amylopectin can happen during frozen storage (Jiang et al., 2021). During freezing, ice crystal growth disturbs the smoothness of starch gels, resulting in a spongy structure. This is explained by the compaction of starch by ice crystals, which enhances starch recrystallization. The water-holding capacity of the starch gel goes down, and free water pockets can arise when the gel is thawed. This formation of pockets of free water is called syneresis. Freezing rate is of influence on the size of crystals, and the growth speed of amylose crystals (Álvarez et al., 2005). The gelatinized starch inside the potato cells is assumed to behave similarly to starch gels during freezing. Waxy potato starch varieties have a lower amount of amylose starch, and they show very little syneresis after freeze/thaw cycles (Jobling et al., 2002).

One should mind that thawing is often not relevant to French fries manufacturing, as they are usually finish-fried from the frozen state. The retrogradation during freezing can modify the firmness of starch gel, and thus the texture of the core (Anna et al., 2019). For starches differing in amylose content, one finds differences in texture due to differences in retrogradation (Reeve, 1967). Also, sugars produced during storage of the raw potatoes modulate the retrogradation (Reeve, 1967) (Allan and Mauer, 2022). Also, we note that retrograded starch can be viewed as resistant starch, lowering glycemic index (Farhat et al., 2001). More resistant starch is formed during freezing as the compaction of starch gel by ice crystals promotes retrogradation of amylose (Raigond et al., 2015).

## 4 Available knowledge

In the previous section, we discussed a variety of physicochemical factors that influence the final texture of frozen par-fried French fries. Fig. 3, Fig. 4 illustrate the intricate causal networks formed by these factors. Additionally, these causes link to different organizational levels of the food product's tissue, as depicted in Fig. 2. We believe that understanding the interplay of these factors requires mathematical modelling. Due to the multiscale nature of these causal factors, their mathematical description necessitates a multiscale modelling approach.

Consequently, in this section, we review the state of the art in the mathematical description of all physicochemical processes underlying the said causal factors. For clarity, we state that our discussion is restricted to only first-principles models, because we think also these models can embody the above discussed causal relations.

**Fig. 5:**
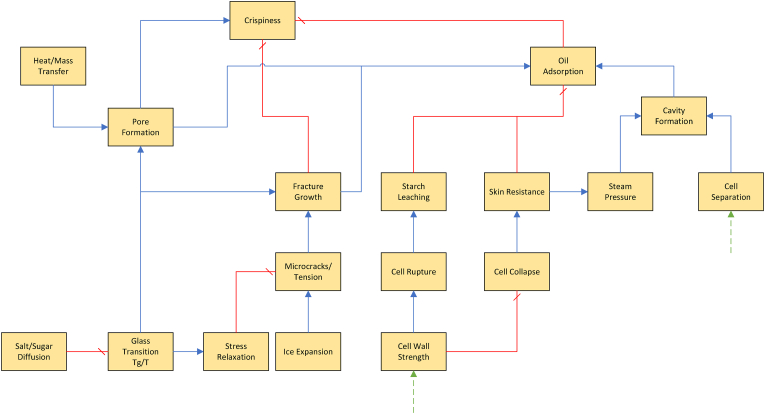
Coupling of submodel at multiple scales for explaining crust crispiness. Green dashed arrows indicate coupling to models, described in Fig. 6. (For interpretation of the references to color in this figure legend, the reader is referred to the Web version of this article.)

**Fig. 6:**
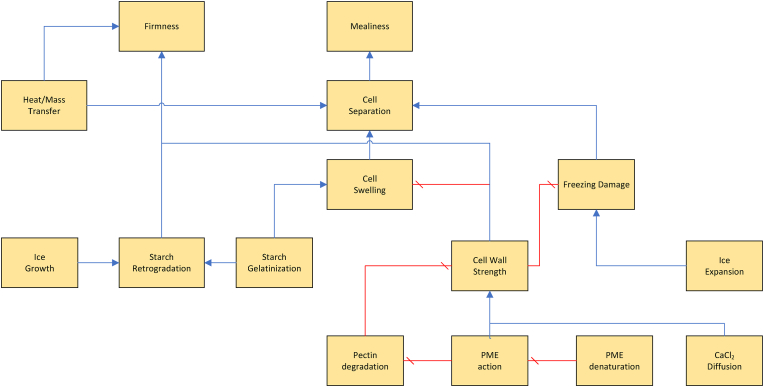
Coupling of submodel at multiple scales for explaining core mealiness/firmness.

For the overview of the reader, we have summarized our findings in Table 5, Table 6, where we have also included a column indicating our view on the complexity of the mathematical model, which can serve as a guide for the reader interested in pursuing a mathematical description of the final texture. For some factors there are no models available yet, which will be indicated with empty entries in the table. Consequently, we rate these models as highly complex. Finally, in Fig. 5, Fig. 6 we have indicated the multiscale coupling between the different models. At the end of the section we briefly describe the required nature of the multiscale coupling.

**Table 5:** Knowledge sources for (possible) causes of external texture.

(Possible) cause	Models	Ref.	Complexity
**Molecular level**			
Moisture transport	moisture diffusion/*a*_*w*_	van der Sman and Meinders (2013)	+
Glass transition	Couchman-Karasz/State diagram	Van der Sman and Meinders (2011)	+
Crust strength/hardness	viscoelastic model	Van der Sman et al. (2022)	+
**Cellular level**			
Case hardening	–	–	++++
**Microstructural level**			
Porosity/structure	cell model	Van der Sman and Broeze (2014b)	+++
Oil adsorption	pore network	Vauvre et al. (2014)	+++
**Crust level**			
Stress development	continuum model	Tremeac et al. (2007)	++
Crust thickness	continuum heat/mass transfer	(Van Koerten et al., 2017) (Gouyo et al., 2021b)	++

**Table 6:** Knowledge sources for (possible) causes of internal texture.

(Possible) cause	Models	Ref.	Complexity
**Molecular level**			
Pectin chemistry	Kinetic model	Verlinden and De Baerdemaeker (1997)	+
PME activity	Kinetic model	Moens et al. (2021), van Dijk and Tijskens (2003)	+
Syneresis	WHC model/Flory Rehner	Van der Sman et al. (2013)	++
Starch gelatinization	Flory-Huggins	Van der Sman and Meinders (2011)	+
Starch retrogradation	Lauritzen-Hoffman	Farhat et al. (2000)	+
**Cellular level**			
Cell swelling	hyperelastic model	Van der Sman (2015b)	++
Cell separation	FEM/SPH-DEM	Paul Van Liedekerke et al. (2010), (2011), Mihai et al. (2018)	++++
Cell wall strength/stress	Cell model	Van der Sman (2015b)	++
Starch swelling	Cell model	Van der Sman and Meinders (2011)	++
**Microstructural level**			
Core structure	Multiscale	–	++++
Ice formation	Phase field	Van Der Sman (2016)	+++
Tissue Damage	Multiscale	–	++++
**Core level**			
Damage/fractures	Continuum/Multiphase	Jin and van der Sman (2022)	+++
Heat/Mass transfer	Continuum/Multiphase	Gulati and Datta (2015)	++

### 4.1 External texture

#### 4.1.1 Molecular level: glass transition

In a comprehensive review of the frying process (Vitrac et al., 2000) it is recommended to use a state diagram for understanding the frying process, as is also highlighted in refs. (Van der Sman and Broeze, 2014a, 2014b). This approach proved valuable in explaining the formation of porous and crispy potato snacks. In the state diagram one plots the phase transitions and glass transition as function of temperature and moisture content. Plotting the path of the fried food material in this diagram enables the understanding of the roles of phase/state transitions during the processing. As starch is the main component in French fries, we advise to base the state diagram on starch, which has been predicted by the use of Flory-Huggins theory and Couchman-Karasz theory (Van der Sman and Meinders, 2011). As the French fry has a distinct crust and core texture, one should plot the state of crust and core as separate paths, as in the case of bread (Bernard et al., 2003). Knowledge of *T*_*g*_ is also important in relation to viscoelastic properties. For several biopolymers we have shown that the elastic modulus, viscosity, and viscoelastic relaxation times are a function of *T*_*g*_/*T* (Van der Sman et al., 2022) (van der Sman et al., 2023).

#### 4.1.2 Molecular scale: moisture transport

Describing moisture transport involves constitutive relations for both the driving force (water activity) and the transport coefficient (diffusion coefficient). The water activity of starch and cell wall material can be explained using Flory-Huggins theory (Van der Sman and Meinders, 2011) (Jin et al., 2014). However, stress develops in the drying crust, and thermodynamics requires accounting for the stress in the driving force, as seen in Flory-Rehner theory (Van der Sman et al., 2013) (Van der Sman, 2015a) (van der Sman, 2023b). However, these elastic stresses relax if the crust remains relatively moist, as indicated by the viscoelastic properties of starch (Van der Sman et al., 2022). A predictive theory for the moisture diffusion coefficient is developed for hydrophilic food materials (van der Sman and Meinders, 2013) (Perdana et al., 2014) (Siemons et al., 2019), though we question its predicted values in the glassy state (van der Sman, 2023b). These theories on moisture transport are equally applicable to the wet core.

#### 4.1.3 Molecular scale: solute diffusion

If blanching is performed in hot water, soluble nutrients can leach from the vegetable. Solute leaching from potato tissue is modelled by (Gekas et al., 1993). Such models also give insight into the rate of diffusion during impregnation processes, such as the infusion of CaCl_2_ or the leaching of sugars or salts from/to the crust region as happens during the glucose/SAPP (sodium acid pyrophosphate) soaking after blanching.

For the diffusion of solutes like salt and sugars, one can use the generalized Stokes-Einstein relation if they are diffusion through the starchy matrix or dense cell walls. The generalized Stokes-Einstein relation involves the viscosity of the matrix, which is known for starchy materials (Van der Sman et al., 2022). For diffusion in water-filled intercellular space, one can use the same relation, but insert the viscosity of water.

#### 4.1.4 Microstructural level: cell collapse

The collapse of cells in the crust during slow pre-drying or par-frying can lead to case-hardening. A mechanical model at the cellular/tissue level is developed by (Karunasena et al., 2015), using the Smoothed-PArticle-Hydrodynamics/Deiscrete Element Method (SPH/DEM) method in 2D. Case hardening itself was not modelled, but imposed via constraining moisture levels of the top layer of cells to low values. The effect of case hardening of underlying tissue was investigated. Effects of cell separation are not accounted for, while cell adhesion is implemented. This should be modified upon temperature changes, cf. above kinetic models. In a follow-up model, pores of similar size as cells are introduced, which develop during drying (Karunasena et al., 2015). Subsequently, it was incorporated in a multiscale model (Wijerathne et al., 2019), which is a coarse-grained particle-based (SPH) model. A 3D version is currently under development (Rathnayaka et al., 2020). The disadvantage of such a particle-based multiscale model implementation is the difficult coupling to more traditional continuum/multiphase models, as developed by Datta (Gulati and Datta, 2015; Gulati et al., 2016; Shi et al., 1998). , which are proposed to model heat and mass transfer at product level scale (i.e. crust and core level).

#### 4.1.5 Microstructural scale: pore development

The crust is expected to show largely a closed pore structure (Vauvre et al., 2014). Hence, we expect that the pore in the crust develops in a similar way as in potato snacks, and thus it can be described in a similar manner (Van der Sman and Broeze, 2014b). For pore development in fried potato snacks we have used a cell model, where steam pressure is balanced by the viscosity of the matrix. Steam pressure is governed by the local water activity, which follows Flory-Huggins (Van der Sman and Meinders, 2011). The viscosity of the starchy matrix follows our previous model, showing that it scales with *T*_*g*_/*T* (Van der Sman et al., 2022). But, we think the cell model must be modified with viscoelastic stresses instead of purely viscous stresses, cf. (van der Sman, 2023b). This cell model must be coupled to a continuum model, supplying temperature and large scale moisture transport, cf. (Gulati and Datta, 2015).

Such a continuum model has been developed for potato (Gulati and Datta, 2015), which even deals with case hardening. Case hardening occurs if the cellular materials get near the glassy state. The development of a strong, elastic skin has major effects on the development of porosity during the drying of plant cellular tissue, as shown with a multiphysics model incorporating large deformation mechanics (Jin and van der Sman, 2022). A similar phenomenon is also observed during the drying of seeds (Bai et al., 2019), drying of maltodextrin droplets (Siemons et al., 2020, 2022), and drying of potato tissue (Santiago et al., 2021). We think in French fries this mechanism leads to development of cavities rather than small pores, as shown by the appearance of blisters and cavities when a dense skin is formed (Van Loon et al., 2007).

Some researchers started developing multiscale models for drying of fruits/vegetables (van der Sman, 2022), where shrinkage due to drying is included using continuum large deformation mechanics models, which are coupled to a Representative Elementary Volume (REV) model (Kevin et al., 2020). The latter accounts for changes at the cellular level (due to loss of turgor).

#### 4.1.6 Microstructural level: cavity formation

During frying of frozen par-fried potato strips significant fractures (denoted above as hollowness) develop during finish-frying, and in particular just beneath the crust region (Gouyo et al., 2021a). These fractures/pores develop further upon continued moisture removal. These fractures enable a second mechanism of oil adsorption if ice is still present during finish-frying (Touffet et al., 2020). It is hypothesized that there is a progression of two fronts: a melting front, and an evaporation front. If a fracture connects these two fronts, it drives a gas pressure-driven flow, due to differences in saturated vapour pressure under boiling/freezing conditions, with condensation of vapour at the melting front. The suction of gas towards the melting front allows for capillary suction of oil in the first 2 min of finish-frying. If the melting front disappears this extra suction disappears too. We hypothesize that the existence of these two fronts leads to the clear development of three regions, as defined by (Gouyo et al., 2021a, 2021b): a stiff, dry crust region, a very porous/fractured region beneath the crust region, and a core, with little fractures and less freezing damage to tissue. Upon creation of a crust in the glassy regime, the crust will not shrink anymore (as observed by X-ray tomography (XRT) (Gouyo et al., 2021a)), and continued moisture removal occurs in the region between the evaporation front and melting front, with pore/fracture development - as explained by mechanics in (Jin and van der Sman, 2022). The shrinkage of the stiff crust renders an under-pressure (tensile stress) in the crumb, leading to the expansion of pre-existing pores (at cell junctions, which are enlarged by cell separation).

#### 4.1.7 Microstructural scale: fracturing

Modelling of actual fracturing of a porous structure is challenging. Some progress has been made with the phase field approach for fracturing, but it has not been applied to food materials (Carlsson and Isaksson, 2019). Models applied to amorphous glassy polymers seem most relevant (Dal et al., 2022). This phase field can be coupled to general large deformation finite element models (Arash et al., 2022).

The fracturing of the crust during eating can be understood at a coarse-grained level via an energy balance (Luyten and Vliet, 2006). The total energy supplied by the deformation is 1) stored as elastic energy, 2) dissipated via viscoelastic relaxation of stresses, or 3) via fracturing. If the crust is in a glassy state, the energy can hardly be dissipated viscoelastically, and thus dissipation only occurs via fracturing. The energy to fracture a wall between pores of the crust depends on the ratio of wall thickness and pore radius (*t*/*R*), the elastic modulus of the composite, which depends on porosity via the Gibson-Ashby relation, and the composition of the wall (mainly the moisture content) (Sozer et al., 2011). The latter study claims these relations hold for solid foams in general, and thus also for crusts of fries.

#### 4.1.8 Crust level: heat and mass transfer

Fast heat and mass transfer are required to develop a porous crust during frying (Van Koerten et al., 2015). Heat and mass transfer are strongly coupled, as the transferred heat is largely used to evaporate water at the crust/core interface. The intensive heating creates a sharp boundary between them, allowing the frying process to be approximated as a moving boundary problem (Farkas et al., 1996).

More realistic physical models describing heat and mass transfer during frying are developed by (Van Koerten et al., 2017) (Lioumbas et al., 2012), where the heat transfer coefficient is made dependent on mass transfer (which promotes bubbling and thus convection in the frying oil). Another outstanding study is (Gouyo et al., 2021b), which considers frying frozen par-fried potato strips. In their model three zones are distinguished: crust, intermediate porous zone, and core. It is stated that the overall shrinkage of frozen strips is small, leading to porosity development in the intermediate porous zone, if the crust zone has reached the glassy state. The core region remains without pores. However, this model was applied to hot-air frying, but it can be adapted to oil frying.

### 4.2 Internal texture

#### 4.2.1 Molecular scale: pectin chemistry

Much of the interplay between various (bio)chemical processes influencing the cell wall has been modelled (Verlinden and De Baerdemaeker, 1997) (van Dijk and Tijskens, 2003). These kinetic models describe a) the breakdown via beta-elimination of pectin, b) the action of PME enzyme on the removal of methyl groups (making pectin insensitive to thermal breakdown), and c) the thermal inactivation of PME enzyme. The model is implemented for carrots, but the model structure probably applies to potatoes as well. The model was validated with the rupture stress of carrot tissue. Residual PME activity in blanched potatoes is investigated by (Gonzalez-Martinez et al., 2004), which can provide parameter values for a similar model for potatoes. The above-mentioned model lacks the action of calcium. This information might be obtained from studies like (Son Vu et al., 2006), where the pectin breakdown is investigated for carrots with the use of acids, or Ca^2+^ chelators. A similar analysis is performed on carrots subjected to Ca^2+^ infusion (Sila et al., 2005), which shows a strong correlation between tissue firmness and degree of methylation of pectin. A more simple model for pectin degradation, but also under other pH conditions, is described by (Fraeye et al., 2007). PME activity/inactivation in potatoes is modelled (Tijskens et al., 1997). PME inactivation in potatoes is also studied in (Moens et al., 2021), showing there are two isomers of PME, one more thermolabile than the other. Combined with more sophisticated models as by (Verlinden and De Baerdemaeker, 1997) (van Dijk and Tijskens, 2003), effects of Ca^2+^ crosslinks can be added.

#### 4.2.2 Molecular scale: enzyme kinetics

One of the functions of blanching is to inactivate enzymes that can otherwise cause discoloration or off-flavours during frozen storage. For potatoes PPO is important. The inactivation of PPO in potatoes is investigated in (Anthon and Barrett, 2002). Inactivation kinetics of other enzymes during blanching is discussed by (Reis, 2017). With the knowledge of enzyme inactivation, one can compute the amount of desired heat input for blanching, which needs to be controlled for the core texture, as pectin degradation happens in this unit operation.

#### 4.2.3 Molecular scale: starch gelatinization

Also, starch gelatinization happens during blanching - which is a factor in cell swelling. A simple first-order kinetic model of starch gelatinization in potatoes, with Arrhenius temperature dependency, is developed by (Verlinden et al., 1995). This model is linked to an energy balance, based on Fourier law for heat conduction.

#### 4.2.4 Molecular scale: starch retrogradation

Starch retrogradation can be modelled via Lauritzen-Hoffman theory (Farhat and Blanshard, 2018). This retrogradation increases crosslinks in the starch matrix, which can lead to the syneresis of water upon thawing, also impacting the water holding capacity (WHC). Effects of crosslinks on WHC follow from Flory-Rehner theory (Van der Sman et al., 2013).

#### 4.2.5 Molecular scale: cell wall structure

Molecular level models of the structure of cell wall materials are reviewed by (Cosgrove, 2014). We view that these molecular models are too detailed for obtaining an understanding of the influence of structure on the texture of fries. In our view coarse-grained models are more appropriate, as described in (Veytsman and Cosgrove, 1998) (Van der Sman, 2015b) (Huang et al., 2015).

#### 4.2.6 Cellular level: cell swelling

Cell swelling occurs if intracellular starch gelatinizes. As similarity between swelling pressure and turgor pressure is indicated, we think that cell swelling can be modelled similar to our earlier hyperelastic model, where we described plant cell volume as function of turgor pressure and cell wall stiffness (Van der Sman, 2015b). Swelling pressure of starch follows Flory-Rehner theory (Jarvis et al., 1992) (Desam et al., 2018), which was also the basis for our hyperelastic model (Van der Sman, 2015b).

By combining the above-described models we can describe the starch gelatinization and swelling inside a potato cell, with a given cell wall strength. Cell wall strength needs to be related to actions of pectin degradation or strengthening by calcium, as discussed in the above paragraph on pectin chemistry. The action of calcium on cell wall strength is described in (Ptashnyk and Allen, 2018), showing how it impacts the large deformation of cells. Their model uses a similar large deformation framework as in our previous work (Van der Sman, 2015b).

#### 4.2.7 Cellular level: adhesion/cell separation

Cell separation depends on the rounding of cells via the swelling pressure, and the adhesion between cells, provided by the middle lamella - as provided by the calcium bridges between pectins of both cells. Cell adhesion is accounted for in the model of the mechanics of cellular tissue of ripened apples (Paul Van Liedekerke et al., 2011), which uses a SPH/DEM multiscale modelling technique, which is a rather computational intensive technique. A more feasible model of debonding between adhering cells is described in (Mihai et al., 2018), which follows the more commonly used Finite Elements Method (FEM). Here, cells are approximated as 2D-hexagonal units, with fiber-reinforced cell walls, and internal pressure (due to turgor).

#### 4.2.8 Microstructural level: tissue strength

A mechanical constitutive model for large deformations of *raw* potato tubers is developed in (Böl et al., 2020). Keep in mind that for raw potatoes turgor is dominating the mechanical response. Because of the parallel between turgor pressure and swelling pressure of gelatinized starch, this model is of interest. However, in cooked potato tissue the mechanics is also imparted by the cell separation.

Similar continuum-level mechanical models (assuming tissue is a homogeneous material) are also developed for drying plant foods, as reviewed by (Mahiuddin et al., 2018). Some of these models are applied to potato (Gulati et al., 2016).

#### 4.2.9 Microstructural level: freezing damage

In the study (Feng et al., 2022) it is shown that freezing of artificial cell walls, composed of pectin and xyloglucan hemicellulose, promotes crosslinking (via compaction of cell wall material). In the absence of Ca^2+^, hydrogen-bonded crosslinks between pectin and xyloglucan are stimulated via the compression of ice crystals. In the presence of Ca^2+^ the strength of the ionic network induced smaller ice crystals, and less freezing damage. A (strong) elastic network makes nucleation more difficult (Hasan et al., 2021). This irreversible compaction of cell wall material is also observed after freeze-drying and rehydration of vegetables (carrots) (Adrian et al., 2012).

The stresses due to the volumetric expansion of ice formed during the freezing of food material are modelled in (Shi et al., 1998). The follow-up study (Shi et al., 1999) shows the possibility of crack formation due to fast, deep freezing. The later extension of this model to two-layer foods (Tremeac et al., 2007) (Ben Aissa et al., 2008) is more relevant for French fries, having distinct crust and core regions. Recent modelling has shown that crust can remain largely unfrozen during freezing operations (van der Sman, 2023a). Freezing damage of foods, in general, is reviewed in (Dalvi-Isfahan et al., 2019). Furthermore, the growing ice crystals lead to compaction of the starch matrix, which can enhance its further retrogradation (Jiang et al., 2021). This might have a negative influence on texture.

#### 4.2.10 Core level: heat and mass transfer

During finish-frying there is significant moisture loss also from the core. This will lead to cell shrinkage, and further cell separation, increasing mealiness and firmness. Heat and mass transfer in the porous core can be modelled in a similar manner as for the crust, cf. (Gulati and Datta, 2015). Mind, that the core starts in the frozen state when finish-fried. Hence, the heat-transfer model needs to account for the ice melting, cf. (Gouyo et al., 2021b).

### 4.3 Multiscale coupling

The type of coupling of multiscale models depends on the separation of length and time scales (van der Sman, 2022). As indicated in Fig. 5, Fig. 6 the length scales of molecular scale, cellular scale and tissue scale can be well separated. However, the timescales of various molecular processes will be at similar time scales as the heat and mass transfer. The molecular processes can even determine processing times like that of blanching (Van der Sman, 2020). Enzyme kinetics, starch gelatinization and cell wall dissolution happen at time scales similar or larger than the heat/mass transfer. Hence, the models at different length scale must be solved with similar time steps, requiring parallel coupling (van der Sman, 2022). But, models at the microstructural level can work with Representative Volume Element models, such as the cell model in ref. (Van der Sman and Broeze, 2014b). Models at the molecular scale do not require resolution of spatial gradients, and thus ordinairy differential equations or thermodynamic relations will suffice (van der Sman, 2022).

## 5 Measurements

In this section, we distinguish measurement methods for a) texture, and b) their physical causes. The methods for texture measurements are already quite mature. Here, we first focus on measurement methods for physicochemical causes of textural variations, which we will subdivide into classes, as determined by the physical length scale they probe. We distinguish the following length scales: molecular level, cellular level, tissue (microstructural) level, product region level (i.e. crust or core). The measurements of textural properties are evaluated at the largest length scale, i.e. crust and core.

In Table 7, Table 8 we have summarized the discussed methods for measuring physicochemical causes, with references to scientific literature. The measurement techniques are sorted according to the length scales they probe. Often, the measurements probe physicochemical causes, which are also referenced by the models listed in Table 5, Table 6 Hence, these measurement techniques can be used for validation of the models.

**Table 7:** Techniques to identify (possible) causes of external texture.

(Possible) cause	Technique(s)	Ref.	Effort	Value
**Molecular level**				
Moisture profile	MRI	(Esveld et al., 2012) (Isik et al., 2018) (Yang et al., 2019)	++	+++
	T2-NMR imaging	Li et al. (2020c)	+++	+++
	XRT (density)	Watanabe et al. (2008)	++	+++
	Drying oven method		+	+
Glass transition	DSC	(Kasahara et al., 2002) (Rahimi et al., 2017)	+	+?
	DMTA	Rouillé et al. (2010)	+	++
**Cellular level**				
Case hardening	Microscopy	Reeve and Neel (1960)	+	+
	MRI	Ruan et al. (1991)	++	++
**Microstructural level**				
Porosity/structure	XRT (X-ray tomography)	(Van Koerten et al., 2015) (van der Sman et al., 2021) (Van der Sman et al., 2018) (Van Dalen et al., 2007)	++	+++
	Direct immersion method	Kalogianni and Papastergiadis (2014)	+	++?
	UV-VIS CSLM	Achir et al. (2010)	++	++
	light microscopy	Reeve and Neel (1960)	+	+
Corner compactness	SEM	Sadeghi et al. (2021)	++	+
Oil adsorption	T2-NMR/MRI	(Yang et al., 2020) (Isik et al., 2018)	++	++
**Crust level**				
Crust thickness	MRI		++	++
	Image analysis		+	++
Crust strength/hardness	Texture analyzer		+	+

**Table 8:** Techniques to identify (possible) causes of internal texture.

(Possible) cause	Technique(s)	Ref.	Effort	Value
**Molecular level**				
Pectin chemistry	Immunofluorescence	Parker et al. (2001)	+++	++?
	Chemical analysis	(Kita, 2002) (Jaswal, 1969) (Murayama et al., 2017) (Binner et al., 2000)	+	++
PME activity	Assay	Canet et al. (2005)	+	++
Syneresis	LF-NMR	Chen et al. (2020)	++	++
Starch gelatinization	DSC, XRD	Karlsson and Eliasson (2003)	+	++
Reducing sugars	NIR	Escuredo et al. (2021)	+	+
Starch retrogradation	DSC, XRD	(Yang et al., 2019) (Shu et al., 2022)	+	++
Amylose/Amylopectin ratio	SEC, spectroscopy	(Hovenkamp-Hermelink et al., 1988) (Visser et al., 1997)	+	+
**Cellular level**				
Cell separation	Shear rheology		+	+?
	Compression test	(Andersson et al., 1994) (Álvarez et al., 1998)	+	+
	Kramer shear cell	(Walter et al., 2002) (Woodman and Warren, 1972)	+	+
Cell adhesion	Microfluidic shearing	Atakhani et al. (2022)	++	++?
	Microscopy + osmosis	Atakhani et al. (2022)	++	++
	Micromanipulators	Atakhani et al. (2022)	+++	++?
	90° peel test	Atakhani et al. (2022)	+	+?
	Cell cohesion test	Segonne et al. (2014)	+	++?
Cell wall strength	AFM	(Zdunek and Kurenda, 2013) (Kirby et al., 1996)	+++	+
	Amylase digestion	Ding et al. (2019)	+	+?
Starch swelling	Osmotic dehydration	Jarvis et al. (1992)	+	++
Cell size	Laser diffraction	Innings et al. (2020)	+	++
Cell stress	FTIR imaging	Jarvis and McCann (2000)	+++	++?
Cell strength	Microindentation	Zafeiri et al. (2021)	++	++
**Microstructural level**				
Core structure	CSLM	Yang et al. (2019)	++	++
	SEM	Yao et al. (2021)	++	+
	Dielectric spectroscopy	(Lewicki and Pawlak, 2005) (Andersson et al., 1994) (Fuentes et al., 2014)	++	++?
Ice formation	XRT	(Vicent et al., 2019) (Mousavi et al., 2007) (Adrian et al., 2012)	++	++
	NIR/microslicing	(Do et al., 2015) (Mishra et al., 2022)	+++	++?
Tissue Damage	Electrical impedance	Ando et al. (2016)	+	++?
**Core level**				
Freezing damage	Uniaxial compression	Valetudie et al. (1999)	+	+
Dry Matter	NIR	(Cees Van Dijk et al., 2002) (Escuredo et al., 2021)	++	+
Water holding capacity	Centrifugation test	Paudel and Boom (2016)	+	+

Furthermore, in the tables, we have scored these measurement techniques regarding effort, and value. In the value estimation, we sometimes include ’?’ to indicate our uncertainty in the value of the measurement method.

### 5.1 External texture

#### 5.1.1 Molecular level

Measuring moisture migration in crispy materials is challenging, but it can be done using single-point Magnetic Resonance Imaging (MRI) (Esveld et al., 2012). Intensities as measured with XRT scales with the moisture content, as has been shown for dried wood (Watanabe et al., 2008). Hence, XRT may equally be applied to measure moisture distribution in French fries, as is also suggested by the study on vegetable tissue (Santiago et al., 2021).

Differential Scanning Calorimetry (DSC) is used to determine the glass transition of the crust of fried potato products (Kasahara et al., 2002) (Rahimi et al., 2017). DSC is also used to investigate the influence of sugars and salt on *T*_*g*_ (Kasahara et al., 2002). These substances are leached/impregnated to modify crust properties and taste. Furthermore, they also modify the freezing temperature (van der Sman and van den Oudenhoven, 2023), which can also be probed by DSC. *T*_*g*_ can alternatively be measured via Dynamic Mechanical Thermal Analysis (DMTA) (Rouillé et al., 2010), which may give a clearer signal for biopolymers like starch.

#### 5.1.2 Microstructural level

Characteristics of the crust can conveniently be analysed with XRT (Van Koerten et al., 2015). XRT is also used for fried, potato starch snacks to relate the microstructure to the crispiness of fried potato starch snacks (van der Sman et al., 2021) (Van der Sman et al., 2018). Application of sophisticated image analysis techniques to XRT images renders various characteristics of crispy food materials such as pore size distributions, wall thickness, and pore connectivity (Van Dalen et al., 2007).

In the study (Achir et al., 2010) Confocal Laser Scanning Microscopy (CLSM) is used to obtain a 3D image of the microstructure of crust, via which they were able to image pores, oil pockets, cut cells (due to the slicing), cell separation due to starch swelling and/or dehydration. Cell detachment was particularly observed in layers beneath the crust.

An alternative method instead of CLSM microscopy is proposed for a less involved quantification of crust structure: the direct wicking method (Kalogianni and Papastergiadis, 2014). After frying oil is removed via extraction method (e.g. Soxhlet) the advancement of adsorbed-water front is observed with a camera. The crust needs to be separated from the core before the measurement.

Via microscopy (Reeve and Neel, 1960) it is shown that case hardening is via the collapse of tissue at the surface due to drying (before frying). Effects of case hardening are also shown via MRI (Ruan et al., 1991), which indicates that during drying the moisture shows a relatively uniform profile, except for a sharp gradient at the surface. This is explained by the compactness, and small porosity of the collapsed skin - showing that indeed the collapsed skin is relatively impermeable to moisture.

A correlation is shown between oil content with either Time-Domain-Nuclear-Magnetic-Resonance (T_2_ NMR) or MRI intensity (Yang et al., 2020). While MRI gives information over the spatial distribution of protons, NMR gives information over different proton populations - but averaged over the sample. Thus NMR can distinguish oil and water, but MRI can not do that. However, by combining NMR and MRI techniques simultaneously one can image both oil and moisture spatial distributions (Isik et al., 2018). However, this requires some water/oil suppression pulsing techniques. The resolution of MRI is, of course, less than XRT, but renders different information like simultaneous oil/water distribution.

Using MRI, SEM (Scanning Electron Microscopy), and XRD (X-ray diffraction) the effects of freezing are shown on oil adsorption of finished-fried potato strips (Yang et al., 2019). Low Field (LF)-NMR shows oil content distribution, and SEM renders a qualitative insight into the microstructural changes in the crust. XRD showed some crystallinity after freezing (due to amylose retrogradation: as indicated by V-type crystals), which decreased with (finish-)frying time. Surface roughness increases similarly to the increase of porosity (which increases with frying time), contributing to oil adsorption.

#### 5.1.3 Crust level

Traditionally only force/displacement measurements with a texture analyzer are used to measure crispiness. Crispiness and crunchiness are distinguished (Sadeghi et al., 2021). Crispiness is evaluated via texture analysis with a sharp blade. Crispiness is equated to the ratio of force and displacement at the first fracture, while crunchiness is related to the number of fracture events. The maximum force is related to hardness. The crispiness of the crust is shown to correlate better with combined measurements of acoustic emissions and mechanical properties with the texture analyzer (Gouyo et al., 2020). Crispiness/crunchiness is also shown to relate to the number of peaks in either force-displacement curves and/or sound emissions (Miranda and Aguilera, 2006).

### 5.2 Internal texture

#### 5.2.1 Molecular level

Standard analytical techniques are employed to determine the chemical composition of potatoes. This includes assessing total nitrogen, total sugars, reducing sugars, sucrose, non-starch polysaccharides, lignin, total pectins, protopectins, and soluble pectins (Kita, 2002). A similar chemical analysis is used to determine the degree of esterification of pectin and the amount of calcium (Jaswal, 1969; Murayama et al., 2017). Via other chemical tests, one can determine the amount of solubilized pectin from the cell wall via the number of uronic acids (Binner et al., 2000).

Enzyme assays are used for measuring PME activity (Canet et al., 2005). Before the use of the assay, the tissue was disrupted, and the supernatant from centrifugation was collected for analysis.

The state of starch regarding gelatinization or retrogradation can be obtained via DSC, using cellular fragments of potato (Karlsson and Eliasson, 2003).

With DSC and XRD it is shown that starch can retrograde inside the potato cell (thereby lowering its digestibility) (Shu et al., 2022). XRD can distinguish amylose and amylopectin retrogradation.

A rapid spectroscopic technique is available to determine the amylose/amylopectin (AM/AP) ratio in potatoes, via the use of I_2_-KI solution added to extracted cellular fluid using perchloric acid (Hovenkamp-Hermelink et al., 1988). A more common method is to use size exclusion chromography (Visser et al., 1997).

NIR can also be used to measure reducing sugars, with the advantage that it can also be implemented online (Escuredo et al., 2021).

With LF-NMR it is shown that after freeze-thaw cycles free water pools are created, due to retrogradation/compaction of starch during freezing (Chen et al., 2020). This retrogradation is also affecting digestion.

Regions of the cell wall with low- and high-methyl-esterified pectin can be imaged via antibody-(immunogold)-labeling techniques and SEM microscopy (Parker et al., 2001). This can be used to study cell separation.

Moisture content distribution in French fries can be imaged with T2 NMR imaging (Li et al., 2020c). The dry matter of potato is normally measured via the under-water-weight method, or drying oven test. It is correlated with starch content.

#### 5.2.2 Cellular level

It is suggested to use Fourier Transform InfraRed (FTIR) imaging to investigate the mechanical properties of cell walls (Jarvis and McCann, 2000). Despite FTIR being more commonly related to compositional changes in cell wall (Radotić et al., 2012), the response could be correlated to mechanical properties as measured via Atomic Force Microscopy (AFM).

Micromanipulation techniques are used to measure the stiffness of individual (cooked) cells (Zafeiri et al., 2021). After separating individual cells, they were subjected to compression with a microindentator (cylindrical probe with an area much larger than the cell diameter). Stiffness is derived from the slope of force/displacement curves. A similar analysis is performed for tomato cells, but with the use of AFM (Zdunek and Kurenda, 2013). This produces data on a subcellular scale. (Kirby et al., 1996) shows some dehydration is required to image via AFM. It rendered the topology and thickness of (cellulosic) fibers in cell walls. Hence, it is questionable whether this can be of value to overall texture properties, considering the large variation in measurements at the nanometer scale.

The swelling pressure of starch inside cells is measured by subjecting potato cell suspensions to osmotic solutions, making cells swell/shrink (Jarvis et al., 1992). Laser diffraction is used to measure the cell size of a suspension of cells (Innings et al., 2020). This can be translated to potatoes if individual cells can be separated (e.g. via chelation of calcium via citric acid).

Several techniques to measure cell adhesion are reviewed by (Atakhani et al., 2022). Via (micro)fluidic approaches a deposited tissue can be subjected to shear forces induced by fluid flow. A problem with this technique might be that adhesion to the substrate must be stronger than cell-cell adhesion. Perhaps, the deposition of sample into a well in the wall can constrain the tissue sufficiently. Via microscopy imaging techniques the angle between (two) cell assemblies at the junction point can be taken as a measure for adhesion. Via different external osmotic pressures, the cells can (de)swell, and different angles can be shown.

Adhesion at the cellular level can also be investigated via micromanipulators, like micropipets. First, they can be (partially) sucked in, when adhered to each other, and force can be measured to separate the cells from each other.

At the level of cell walls, the strength can be measured via AFM. It can be used for cell adhesion, but one cell needs to adhere to the AFM tip, which is a challenge.

An assay with digestion via alpha-amylase enzyme can indicate the degree of damage to cell wall (Ding et al., 2019).

#### 5.2.3 Microstructural level

Changes in porosity can be measured via electrical conductivity. Sometimes, cells are not separated but fractured through the cell - leading to the leaching of starch (Lewicki and Pawlak, 2005).

Dielectric properties can be used for assessing mealiness, as shown in (Andersson et al., 1994). A related technique is impedance spectroscopy (Fuentes et al., 2014). The major response is due to the loss of cell membrane integrity - which is already lost by the Pulsed Electric Field (PEF) treatment before slicing. But, perhaps the porosity changes due to cell separation also change the impedance.

NIR spectral imaging shows 3D images of ice crystals in frozen beef (Do et al., 2015). This shows potential for NIR imaging of ice in sliced cross sections of French fries via a spectral imaging system.

SEM is used to image cellular structures of fried potato (Yao et al., 2021). However, SEM images are difficult to quantify, because of problems of identification of structures and restricted field of view.

The structure of frozen vegetables (carrots) is investigated with XRT (Vicent et al., 2019). This study means an improvement compared to previous studies, as they were able to image the ice frozen with sufficient contrast difference compared to the unfrozen phase. Previous studies mostly relied on freeze-drying to remove the ice fraction, leaving a gas-filled pore phase, having a large contrast difference with the unfrozen phase (Mousavi et al., 2007) (Adrian et al., 2012). XRT imaging of frozen samples requires special sample holders, which can keep samples frozen, either with phase change materials or with Peltier elements.

XRT imaging (using freeze-drying pretreatment) combined with electrical impedance spectroscopy is used to assess freezing damage to vegetables or sweet potatoes (Ando et al., 2019b) (Lee and Watanabe, 2022). We must remark that before freezing the vegetables were in the raw state, and they had intact cell membranes. In these cases the freezing operation would damage the cell membrane, giving a clear change in the electrical impedance. For industrial processing of French fries, it holds that products are blanched before freezing, meaning that cell membrane integrity is already lost. As discussed above, electrical impedance spectroscopy still might have value if cell separation gives a significant change in the impedance. A study on blanching and freezing pretreatments before drying carrots does seem to indicate changes in impedance after different pretreatments (which all induce loss of cell membrane integrity) (Ando et al., 2016).

Cell size and swelling might also be inferred via water holding capacity measurements using centrifugation (Paudel and Boom, 2016). This technique has not been applied to potatoes (tissue) but to other vegetable tissues like mushrooms and carrots.

#### 5.2.4 Core level

Mealiness can be measured with a texture analyzer with Kramer Shear Cell probe (Andersson et al., 1994) (Walter et al., 2002) (Woodman and Warren, 1972).

A cell cohesion test has been developed for assessing mealiness in apples (Segonne et al., 2014). Cylindrical samples are taken from the tissue, and subjected to shearing forces by submerging the samples in a vortexed fluid. A dye is added to the fluid to enhance the microscopic identification of cell density in the decanted fluid. A reasonable correlation between the cell cohesion test and sensory scores of mealiness is obtained. Such a test can be adapted to (fried) potatoes.

The Texture Profile Analysis (TPA) protocol for texture analyzers gives a good correlation with firmness or hardness (Leung et al., 1983), but correlates poorly with mealiness in cooked potatoes.

Various rheological methods (compression/tension/shear/creep tests) are investigated to correlate to texture (Álvarez and Canet, 1998). For the tensile test, tissue needs to be intact and shaped into dog bones, which limits its applicability. The compression test proved to have the best correlation with texture, combined with a low degree of variation.

A quick test for adhesion at the tissue level is a 90-degree peel test. Some adhesive is attached to the tissue, and with a texture analyser, this is peeled off. Again stronger adhesion to the substrate is required. The method might be prone to large (random) variations in measurements. Nitrocellulose substrates are advised (Atakhani et al., 2022).

Viscoelastic properties of cooked potato tissue are measured via creep test using compression cycles, sometimes in combination with shear in orthogonal direction (Álvarez et al., 1998). This analysis was complicated, due to different contributing factors like loss of turgor, cell wall softening, and starch gelatinization.

Indention tests are proposed for local measurements of mechanical properties (Liu and Scanlon, 2007). Similarly, a single puncture probe with texture analyzer *in-situ* during frying was proposed (Pedreschi et al., 2001). Force is measured as a function of penetration depth. It showed (development) of crust hardness and thickness, and (development) of core softness.

Rheometers are hardly used for measurements of texture. However, a DMTA-rheometer is used to observe crust development in bread material (Vanin et al., 2010), which can be adapted to fried foods.

For individual potato strips, NIR can be used (Cees Van Dijk et al., 2002). NIR correlated with dry matter and perceived texture, it was shown that dry matter varied within one potato. The contribution to the core texture due to cultivar differences is smaller than that of the dry matter (mealiness and graininess).

## 6 Alternative processing affecting physical causes

In this section we discuss the application of novel technologies in different processing steps, or as additional unit processes, and its possible consequence for the final texture. They are discussed in the order they would appear in the production chain.

### 6.1 PEF

Currently, Pulsed Electric Field (PEF) technology is already used in industry to induce loss of turgor before cutting, which then requires less energy and shows less wear on the cutting knives (Botero-Uribe et al., 2017). PEF makes the membranes of the cell and vacuole permeable, allowing passage of water and solutes. Water can migrate to the intercellular space, via relaxation of stress in the stretched cell wall of the permeated cell.

Several other benefits have been claimed for PEF in vegetable processing, but they are strongly criticized in literature (Roman et al., 2013) (Terefe et al., 2015). One of these claims is the selective non-inactivation of enzymes, but like other claimed effects its effect is very likely caused by the thermal input of the PEF process. We think the only other practical value of PEF in the fries production chain is to assist the infusion/impregnation of solutes during impregnation before blanching (like Ca^2+^ and enzymes), due to the increased permeability of cell membrane via the PEF treatment (which already happened before the cutting operation).

### 6.2 Impregnation

It can be beneficial to impregnate the potato tissue with solutes before blanching, which can contribute to overall texture improvement or other quality improvements. It is proposed to use enzymes during impregnation before blanching (Lisińska et al., 2007): pectolytic/hemicellulitic enzymes weaken cells in the crust, allowing for more cell swelling and thus starch gelatinization, leading to cell rupture and subsequent leaching of starch to form a coating preventing oil adsorption. Also, asparaginase can be added to prevent acrylamide formation. Firmness can be improved by impregnation of CaCl_2_ and/or lactic acid before blanching (Jia et al., 2020). The effect of lactic acid might be via the pH lowering, which reduces pectin degradation. Lower pH might also inhibit some enzymes. The effects of lactic acid on taste need to be evaluated. CaCl_2_ infusion promotes the extra formation of crosslinks in de-esterified pectin during LTLT (Long-Time-Low-Temperature) blanching.

Vacuum impregnation process on a whole fresh potato has been investigated (Hironaka et al., 2011). Impregnation was restricted to some surface area, but the impregnation fluid could also penetrate the central region, via the vascular tissue. This poses some opportunity to reach the central region of the potato, where dry matter and cell wall strength might be lower compared to the rest of the tuber. Via vacuum impregnation, one can infuse the tissue with functional ingredients, such as Ca^2+^, or acids - as discussed above. However, for French fries manufacturing, it is still probably beneficial to apply impregnation after the cutting operation, which gives a very significant shortening of the diffusion distance. Vacuum impregnation is applied before blanching of potato chips, which were subjected to microwave-vacuum drying (Barreto et al., 2019). It rendered a more porous structure after drying.

### 6.3 Blanching

LTLT (Low-Temperature-Long-Time) blanching can induce the strengthening of cell walls via the activation of the PME enzyme, and Ca^2+^ crosslinking. We remind the reader that extended blanching is accompanied by more solute leaching. If potato strips contain too much sugar, this can be desired (to reduce the Maillard reaction). In general, a two-step blanching is advised (Moyano et al., 2007), where LTLT is applied prior to HTST (High-Temperature Short-Time), with the latter inactivating PPO enzymes. LTLT is said to be a means to reduce the variability in texture (Agblor and Scanlon, 1998).

Radio Frequency (RF)-blanching can inactivate PPO enzyme at milder conditions, which is said due to better uniformity in heating (Zhang et al., 2018). Less severity of blanching will affect texture via reduced pectin degradation. Ohmic preheating is investigated for its effect on the oil adsorption of fries (Salengke and Sastry, 2007). Ohmic heating can be a fast means of blanching, due to the instantaneous volumetric heating. The texture will benefit from shorter (HTST) blanching times.

### 6.4 Pre-drying

Pre-drying via vacuum-microwave (VM) is investigated (Tajner-Czopek et al., 2008). Here, heating is volumetric, and faster moisture removal is achieved with a lower gradient in moisture content. Consequently, there is less oil adsorption. VM created also a more puffed microstructure, throughout the fry. Also, it can prevent case hardening (Nijhuis et al., 1998).

It is proposed to use osmotic dehydration as a pre-drying method, which might even be combined with blanching (Krokida et al., 2001). Osmotic dehydration gives less case-hardening, and less hardness of crust (via lowering of the moisture gradient). Different osmotic agents can be used like sugars, maltodextrins, or salts. Their influence on other aspects of quality needs to be evaluated. However, still surface water needs to be removed after osmotic dehydration, otherwise splashing of oil will occur during par-frying.

### 6.5 Coating

Also, the use of coatings is proposed (applied before frying), to limit the permeability of crust to oil penetration (Mellema, 2003).

### 6.6 Assisted freezing

Novel non-thermal technologies to assist the freezing process have been reviewed (Jiang et al., 2022). They encompass ultrasound, electro (magnetic) fields, or high pressure. It is concluded that the results of freezing assisted by electromagnetic fields are questionable, as their effect is largely due to volumetric heating to slow down ice crystal growth. Energetically, it is also strange to perform heating and freezing simultaneously.

Ultrasound is said to stimulate early nucleation of ice formation, but ultrasound is only absorbed well if the product is immersed in a fluid. In the case of par-fried fries, this immersion would eliminate the effects of par-frying on moisture loss.

Most of the novel technologies promise to stimulate nucleation and/or formation of small ice crystals. We question whether these crystals remain small during frozen storage, as under these long periods ice crystals can grow via Ostwald ripening.

## 7 Conclusions

From this review, we conclude that integrated knowledge of how all processing steps in the industrial production chain of par-fried frozen fries affect final textural properties is still lacking. This is because often academic studies are restricted to a single step of the production chain. Furthermore, applied pre-treatments are not always conform industrial practice, such as in studies where potato strips are fried from the raw state without blanching or freezing. Moreover, consideration of the complete production chain requires a multidisciplinairy approach.

For the advancement of this required knowledge, we have summarized various physicochemical phenomena happening during manufacturing, contributing to the final quality. These physicochemical causes form a multiscale hierarchy, implying that modelling these phenomena requires a multiscale simulation approach. With the help of causal networks we have shown how these causal factors influence each other, and contribute to the final texture of the crust and core. Their multiscale coupling is also indicated in figures.

Furthermore, we have discussed the currently available theories and models in detail, combined with a discussion of experimental methods probing the occurring physical/chemical phenomena occurring at various scales.

In particular, our overview shows that knowledge of the mechanical behaviour of tissue at the microstructural level is lacking. In mechanics, this field is also still in its infancy, and it requires sophisticated modelling techniques such as SPH-DEM. Furthermore, such models need good validation using multiscale imaging and micromanipulation techniques at the microstructural level.

The ultimate goal of the multiscale model is to provide the industry with a decision support tool to evaluate process innovations for effects on final textural quality, which can be balanced against the impacts of the innovations on sustainability and economics.

## Credits

RGM van der Sman conceptualized the paper, drafted and reviewed the paper. Esther Schenk contributed to the writing of the draft, and reviewed the paper.

## Declaration of competing interest

All authors declare that there are no confllicts of interests.

## Data Availability

No data was used for the research described in the article.
